# Health-Related Quality of Life and Health Utility Values in Beta Thalassemia Major Patients Receiving Different Types of Iron Chelators in Iran

**Published:** 2016-10-01

**Authors:** Meysam Seyedifar, Farid Abedin Dorkoosh, Amir Ali Hamidieh, Majid Naderi, Hossein Karami, Mehran Karimi, Masoomeh Fadaiyrayeny, Masoumeh Musavi, Sanaz Safaei, Mohammad Mahdi Ahmadian-Attari, Molouk Hadjibabaie, Abdol Majid Cheraghali, Ali Akbari Sari

**Affiliations:** 1 Pharmaceutical Management and Economics Research Center, Tehran University of Medical Sciences, Tehran, Iran; 2 Department of Pharmacoeconomics and Pharmaceutical Administration, Faculty of Pharmacy, Tehran University of Medical Sciences, Tehran, Iran; 3 Hematology-Oncology and Stem Cell Transplantation Research Center, Tehran University of Medical Sciences, Tehran, Iran; 4 Department of Pediatrics Hematology-Oncology, Ali-Ebne Abitaleb Hospital Research Center for Children and Adolescent Health [RCCAH], Zahedan University of Medical Sciences, Zahedan, Iran; 5 Thalassemia Research Center, Hemoglobinopathy Institute, Mazandaran University of Medical Sciences, Sari, Iran; 6 Hematology Research Center, Shiraz University of Medical Sciences, Shiraz, Iran; 7 MSc Student of Nursing, Islamic Azad University, Isfahan (Khorasgan) Branch, Isfahan, Iran; 8 Department of Traditional Medicine, Medicinal Plants Research Center of Barij, Kashan, Iran; 9 Research Center for Rational Use of Drugs, Tehran University of Medical Sciences, Tehran, Iran; 10 Faculty of Pharmacy, Tehran University of Medical Sciences, Tehran, Iran; 11 Faculty of Pharmacy, University of Baqiyatallah Medical Sciences, Tehran, Iran; 12 Department of Health Management and Economics, School of Public Health, Tehran University of Medical Sciences, Tehran, Iran

**Keywords:** Thalassemia, Quality of life, Health utility, Iron chelators, Iran

## Abstract

**Background:** Thalassemia is a chronic, inherited blood disorder, which in its most severe form, causes life-threatening anemia. Thalassemia patients not only engage with difficulties of blood transfusion and iron chelating therapy but also have some social challenges and health threatening factors. There are some reports on quality of life in thalassemia patients around the world from southeast of Asia to Italy in Europe and United States. In this study, we tried to evaluate and compare Health Related Quality of life (HRQoL) and the health utility in beta thalassemia major patients receiving different types of iron chelators and living in different socio-economical situations.

**Subjects and Methods:** EQ-5D-3L accompanied by a Visual Analogue Scale (VAS) questionnaire was used. The respondents were patients with beta thalassemia major that were at least 12 years old selected from 3 provinces of Sistan-Blouchestan, Fars and Mazandaran. Comorbidities including heart complication, Diabetes Mellitus and Hepatitis and also types of iron chelators (oral, injection, combination of both) were also asked. Cross tab and ANOVA analysis conducted to evaluate each dimension score and health utility differences between provinces, iron chelation methods, comorbidities, age group and gender.

**Results:** 528 patients answered the questionnaires. The health utility of patients that received oral iron chelator were 0.87 ± .01 for oral iron chelators versus 0.81 ± .01 for injection dosage form (p<0.05). Increase in age was accompanied by decrease in health utility. Females faced more usual activity problems, anxiety and depression. Heart problems were more prevalent in males.

**Conclusion:** This study suggests that the quality of life of beta thalassemia major patients is dependent on type of iron chelation treatment which they received, the gender they have, the comorbidities they suffer and socio-economical situations they live in.

## Introduction

 Thalassemia is a chronic, inherited blood disorder, which in its most severe form causes life-threatening anemia.^1^ Iran is one of the countries located on thalassemia belt, with more than 13000 registered thalassemia major patients.^2^ Management of thalassemia major needs patients to have life-long access to a treatment regimen of regular blood transfusions coupled with iron-chelation therapy.

Adequate chelation can be accomplished by regular use of Deferoxamine infusions, oral take of Deferasirox or combination of injection and oral iron chelators such as Deferiprone or Deferasirox; which are critical for survival of thalassemic patients through reducing the risk of both iron overload and the related life threatening complications.^3^^,^^4^ In most cases, blood transfusions and iron-chelation therapy are started in first years of life and the patients and their families should continue their treatment during their childhood, adolescent and adult years, which causes incompliance for patients and their families.^3^^,^^5^

Thalassemia patients not only engage with difficulties of blood transfusion and iron-chelation therapy but also have some social challenges and health threatening factors.^6^ Despite iron chelation, chronic transfusion therapy often results in severe iron overload and possible dysfunction of the liver, heart and endocrine organs.^7^

By this background, patients’ quality of life (QoL) as the main index of thalassemia treatment outcome should be considered. An evaluation of QoL varies from other medical assessments in which it focuses on the individuals’ own views of their health and assesses other aspects of life, giving a more holistic view of well-being.^8^ There are some reports on quality of life in patients with thalassemia around the world from Southeast Asia to Italy in Europe and the United States; however, the results show similarities in some aspects and differences in others.^9^^-^^12^ These variations are mainly because of cultural and socio-economic differences in target populations which make the concept of quality of life variable.^12^^-^^15^ Decision makers prefer to limit the effect of these differences by using local data. In this study, we tried to evaluate and compare QoL and the health utility in beta thalassemia patients with different situations in Iran using EQ-5D-3L questionnaire and Visual Analogue Scale.

## SUBJECTS AND METHODS

 Health utility score was measured through both direct and indirect methods. Although Visual Analogue Scale (VAS) as a direct method is simple and less cognitively demanding than other direct methods, it does not incorporate time into the health utility score.^8^

To address this shortcoming, we tried to convert the results of EQ-5D to health utility. In this study, the valid Persian translation of EQ-5D-3L questionnaire was used. The respondents were patients with beta thalassemia major aged at least 12 years and were referred during three weeks to the center of Blood Transfusion and Thalassemia Treatment in Ali Asghar Hospital, Zahedan, Sistan*-*Baluchestan*,* Boo Ali Sina Hospital, Sari, Mazandaran and Shahid Dastgheib Hospital, Shiraz, Fars. The socio-economic and cultural factors of these provinces are different with each other.

Each respondent was requested to fill his/her age and gender in the pre-questionnaire sheet and to select one of these condition choices: “with or without heart complication”, “with or without Diabetes Mellitus” and “with or without Hepatitis”. They were also asked to explain the type of iron chelator they use as oral, injectable, or a combination of them. This part of questionnaire was double checked by patients’ caregivers which were also the interviewer.

Then, EQ-5D as a self-report questionnaire was filled by patients. Caregivers were available for any needed guidance. Visual Analogue Scale as a direct tool to measure health utility score was also used. Data were pooled and analyzed by SPSS version 16. Value sets have been derived using US time trade of (TTO) set^16^ algorithm^17^ and Iran TTO set derived by Reza Goudarzi in his thesis entitled “Estimating quality weights for EQ-5D health states with the time trade-off (TTO) method in Iran", 2014. Independent T test and ANOVA followed by Scheffe/Dunnett’s T3 Post Hoc analysis conducted to evaluate each dimension score and health utility differences among provinces, iron-chelation methods, comorbidities, age group and genders.

Description of Terms:

a)    EQ-5D-3L: is a standardized instrument for use as a measure of health outcome developed by Euro Qol.^8^

b)   Visual Analogue Scale (VAS): is a simple measurement for assessing health utility and involves the use of a scale shown on a single line. The top of the scale indicates the ‘best imaginable health’, whereas the bottom of the scale indicates the "worst imaginable health". Individuals are asked to indicate where on the scale they consider the health state of interest to be.^8^

c)   Time Trade Off (TTO): is a tool used in health economics to help determine the quality of life in a patient or group.^8^

d)   Health utility: utilities are cardinal values that represent the strength of an individual’s preferences for specific health-related outcomes.^8^

## Results

 Of these 542 participants, 512 (94%) answered main part of the questionnaires, correctly. The rest of the patients were excluded from the study because of their incomplete and ambiguous answers. Mean age of the respondents was 22 years and 63 people were older than 30 years. Gender contribution was nearly equal (255 males/257 females). A majority of patients used injectable dosage form of iron chelators (n=214), followed by oral or combination of oral and injection dosage (147 oral/151 combination).

Demographic and disease characteristics of respondents are shown in Table 1.

Mean health utility scores, average percentage of Visual Analogue scale (VAS) results, mean scores of each dimension of EQ-5D questionnaire for each group and total health utility of the patient according to US and Iran TTO value set are shown in Table 2 and 3. The health utility of patients that received oral iron chelator was 0.87 ± .01 based on Iran TTO value set and 0.88 ± .01 based on US TTO value set for oral iron chelators, compared to 0.81 ± .01 and 0.84 ± .01 for injection form. The health utility of thalassemic patient according to VAS was 74%. Table 2 and 3 present the results of health utility and its subscales in detail. In Table 2, mean health utility scores are calculated based on Iran value set and in Table 3 they are calculated based on US value set. The patients may have one, two, or three comorbidities at the same time. Thus, the calculation of comorbidity has been done separately.

## Discussion

 EQ-5D-3L is one of the most commonly used standardized health related quality of life (HRQoL) questionnaires and has been used to measure the outcome of treatment for various diseases. It is short, flexible, easy to use and can generate a single total score based on population-based preference weights of HRQoL. It is accompanied by a Visual Analogue Scale (VAS) which, in addition to the results of the questionnaire, can be used to measure health utility score^18^. Health utility score is one of the requirements of cost-health utility analysis in pharmacoeconomic studies.

In this study, the relationship between underlying factors and the quality of life and health utility of patients with beta thalassemia major was assessed. 

Our result suggests that the quality of life in patients with beta thalassemia major is dependent on the type of iron chelation treatment they receive, the gender they have, the comorbidities they suffer, socio-economic situations they live in and their age.

Types of iron chelators had a significant effect on health utility and its subscales. Patients who received the oral dosage form were in better health condition in total. Their health utility score was significantly greater than other groups (who receiving injectable dosage form or a combination of both types).

Their better mobility, lesser pain and discomfort, lesser anxiety and depression were the reasons of this difference. In the same line, previous studies have shown the similar effect of oral dosage form on health utility and HRQoL.^19^^-^^22^

**Table 1:** Demographic and disease characteristics of respondents

		**Heart complication**			**Diabetes**			**Hepatitis**		
		**With**	**Without**	**ND** *	**With**	**without**	**ND**	**with**	**Without**	**ND**
**Gender**	**Female**	72 [28%]	185 [72%]	-	37 [15%]	220 [85%]	-	15 [6%]	239 [92%]	3
	**Male**	109 [43%]	145 [57%]	1	39 [15%]	216 [[85%]	-	15 [6%]	236 [92%]	4
**Provinces**	**Mazandaran**	23 [18%]	102 [81%]	1	26 [20%]	100 [79%]	-	13 [10%]	108 [85%]	5
	**Sistan-Bluchestan**	141 [49%]	148 [51%]	-	35 [12%]	253 [87%]	-	12 [4%]	276 [95%]	1
	**Fars**	17 [17%]	80 [82%]	-	14 [14%]	83 [85%]	-	5 [5%]	91 [94%]	1
**Types of Iron Chelators**	**Oral**	27 [18%]	120 [82%]	-	8 [5%]	139 [95%]	-	7 [5%]	139 [95%]	1
	**Injection**	91 [42%]	122 [57%]	1	43 [20%]	171 [80%]	-	17 [8%]	194 [90%]	3
	**Combination**	63 [41%]	88 [58%]	-	25 [16%]	126 [83%]	-	6 [4%]	142 [94%]	3
**Age group**	**10-15**	21 [28%]	54 [72%]	-	2 [2%]	73 [97%]	-	0 [0%]	75 [100%]	-
	**15-20**	44 [40%]	66 [60%]	-	14 [12%]	96 [87%]	-	6 [5%]	104 [94%]	-
	**20-25**	55 [37%]	93 [63%]	1	17 [11%]	132 [88%]	-	8 [5%]	141 [94%]	-
	**25-30**	36 [33%]	74 [67%]	-	24 [22%]	86 [78%]	-	8 [7%]	100 [91%]	2
	**Over 30**	23 [36.5%]	40 [63.5%]	-	18 [29%]	45 [71%]	-	7 [11%]	51 [80%]	5

* : Not Determined

**Table 2:** Mean health utility scores based on Iran values

**Iran values**		**VAS [%]**	**Total Health** **Utility**	**Mobility**	**Self Care**	**Usual** **Activity**	**Pain and** **Discomfort**	**Anxiety/** **Depression**
**Gender**	**Female**	72.9 ± 1.1	.81 ± .00**	.98 ± .00	.99 ± .00	.97 ± .00**	.96 ± .00	.94 ± .00***
	**Male**	72.6 ± 1.1	.85 ± .00**	.98 ± .00	.99 ± .00	.98 ± .00**	.96 ± .00	.96 ± .00***
**Provinces**	**Mazandaran**	75.3 ± 1.7	.81 ± .01	.98 ± .00	.99 ± .00	.98 ± .00**^a^	.95 ± .00**	.94 ± .00*^a^
	**Sistan-Baluchestan**	72.4 ± 0.9	.83 ± .01	.98 ± .00	.99 ± .00	.97 ± .00**a,b	.97 ± .00**	.96 ± .00*a,b
	**Fars**	69.8 ± 2.2	.83 ± .01	.98 ± .00	.99 ± .00	.99 ± .00**^b^	.96 ± .00	.94 ± .00*^b^
**Age group**	**10-15**	73.5 ± 2.0	.84 ± .01	.98 ± .00	1.00 ± .00	.97 ± .00*	.96 ± .00	.96 ± .00*^a^
	**15-20**	71.7 ± 1.6	.85 ± .01	.98 ± .00	.99 ± .00	.98 ± .00	.96 ± .00	.96 ± .00*b,c
	**20-25**	72.0 ± 1.5	.82 ± .01	.98 ± .00	1.00 ± .00	.98 ± .00	.96 ± .00	.94 ± .00*^c^
	**25-30**	73.4 ± 1.8	.84 ± .01	.98 ± .00	.99 ± .00	.99 ± .00*	.97 ± .00	.95 ± .00
	**Over 30**	72.7 ± 2.5	.78 ± .02	.98 ± .00	.99±.00	.98 ± .00	.95 ± .01	.93 ± .01*a,b
**Cardiac Complications**	**With**	68.9 ± 1.3**	.81 ± .01*	.97 ± .00**	.99 ± .00	.97 ± .00**	.96 ± .00	.95 ± .00
	**Without**	74.6 ± .99**	.84 ± .00*	.98 ± .00**	.99 ± .00	.98 ± .00**	.96 ± .00	.95 ± .00
**Diabetes**	**With**	68.0 ± 2.5*	.79 ± .01*	.97 ± .00	.99 ± .00	.97 ± .00	.95 ± .00*	.94 ± .00
	**Without**	73.3 ± .85*	.84 ± .00*	.98 ± .00	.99 ± .00	.98 ± .00	.96 ± .00*	.95 ± .00
**Hepatitis**	**With**	66.0 ± 4.5*	.79 ± .02	.97 ± .00	.98 ± .00	.97 ± .00	.95 ± .00	.95 ± .01
	**Without**	73.0 ± .82*	.83 ± .00	.98 ± .00	.99 ± .00	.98 ± .00	.96 ± .00	.95 ± .01
**Types of Iron Chelators**	**Oral**	75.7 ± 1.65	.87 ± .01*a,b	.99 ± .00*	1.00 ± .00	.98 ± .00	.97 ± .00*	.96 ± .00*
	**Injection**	71.5 ± 1.22	.81 ± .01*^a^	.97 ± .00*	.99 ± .00	.97 ± .00	.96 ± .00*	.95 ± .00
	**Combination**	71.0 ± 1.40	.81 ± .01*^b^	.98 ± .00	.99 ± .00	.98 ± .00	.96 ± .00	.94 ± .00*

* : p-value <0.05,

** : p value <0.01

*** : p-value <0.001.

a, b and c show p-value is related to which comparison

**Table 3:** Mean health utility scores based on US values

**US Values**		**VAS [%]** **[Repeated]**	**Total Health Utility**	**Mobility**	**Self Care**	**Usual Activity**	**Pain and Discomfort**	**Anxiety/** **Depression**
**Gender**	**Female**	72.9 ± 1.1	.84 ± .00*	.97 ± .00	.99 ± .00	.96 ± .00**	.91 ± .00	.91 ± .00**
	**Male**	72.6 ± 1.1	.86 ± .00*	.98 ± .00	.99 ± .00	.98 ± .00**	.92 ± .00	.94 ± .00**
**Provinces**	**Mazandaran**	75.3 ± 1.7	.83 ± .02	.97 ± .00	.99 ± .00	.98 ± .00** a	.88 ± .00**	.90 ± .01*
	**Sistan- Baluchestan**	72.4 ± 0.9	.86 ± .00	.96 ± .00	.99 ± .00	.96 ± .00**a,b	.92 ± .00**	.93 ± .01*
	**Fars**	69.8 ± 2.2	.84 ± .01	.97 ± .00	.99 ± .00	.99 ± .00** b	.91 ± .00	.90 ± .01
**Age group**	**10-15**	73.5 ± 2.0	.85 ± .01	.97 ± .00	1.00 ± .00	.95 ± .00*	.92 ± .01	.94 ± .01*^a^
	**15-20**	71.7 ± 1.6	.87 ± .01	.97 ± .00	.99 ±. 00	.96 ± .00	.92 ± .01	.94 ± .01*^b^
	**20-25**	72.0 ± 1.5	.84 ± .01	.97 ± .00	1.00 ± .00	.97 ± .00	.90 ± .01	.91 ± .01
	**25-30**	73.4 ± 1.8	.86 ± .01	.97 ± .00	.99 ± .00	.98 ± .00*	.93 ± .01*	.91 ± .01
	**Over 30**	72.7 ± 2.5	.81 ± .02	.97 ± .00	.98 ± .00	.97 ± .00	.86 ± .01*	.88 ± .01*a,b
**Cardiac Complications**	**With**	68.9 ± 1.3**	.83 ± .01*	.96 ± .00**	.99 ± .00	.95 ± .00**	.90 ± .00	.92 ± .00
	**Without**	74.6 ± .99**	.86 ± .00*	.97 ± .00**	.99 ± .00	.97 ± .00**	.91 ± .00	.92 ± .00
**Diabetes**	**With**	68.0 ± 2.5*	.82 ± 0.01*	.96 ± .00	.99 ± .00	.96 ± .00	.88 ± .00*	.90 ± .01
	**Without**	73.3 ± .85*	.86 ± 0.00*	.97 ± .00	.99 ± .00	.97 ± .00	.91 ± .00*	.92 ± .00
**Hepatitis**	**With**	66.0 ± 4.5*	.82 ± .02	.96 ± .00	.98 ± .00	.96 ± .01	.89 ± .02	.92 ± .01
	**Without**	73.0 ± .82*	.85 ± .00	.97 ± .00	.99 ± .00	.97 ± .00	.91 ± .00	.92 ± .01
**Types of Iron Chelators**	**Oral**	75.7 ± 1.65	.88 ± .01*a,b	.98 ± .00*	1.00 ± .00	.97 ± .00*	.93 ± .01*	.94 ± .00*
	**Injection**	71.5 ± 1.22	.84 ± .01*^a^	.96 ± .00*	.99 ± .00	.96 ± .00*	.90 ± .01*	.92 ±.00
	**Combination**	71.0 ± 1.40	.83 ± .01*^b^	.97 ± .00	.99 ± .00	.97 ± .00	.91 ± .01	.90 ± .00*
**Total**		72.5 ± .81	.85 ± .00	.97 ± .00	.99 ± .00	.97 ± .00	.91 ± .00	.92 ± .00

* : p-value <0.05,

** : p-value <0.01,

*** : p-value <0.001.

a, b and c show p value is related to which comparison

Goulas et al. have suggested that patients received Deferasirox (an oral iron chelator) face with less limitation in their ability to work, less annoyance from the length of treatment and in total, higher QoL.^23^ Thavorncharoensap et al. have revealed that injectable iron chelation treatment is significantly related to impair QoL in physical health and school functioning.^24^

In spite of statistical significance, difference of health utility between oral and injectable dosage forms in Iranian patients was lesser than those in other countries. Although health utility score of the oral dosage form measured through TTO in Australia and England was similar to that of Iran (0.93 and 0.84, respectively), this score was lesser than that of Iran for the injectable drug (0.66 for both Australia and England).^19^^,^^21^ It means that consumption of injectable dosage form was more acceptable in Iran, compared to Australia and England. Moreover, Iranian patients who received this kind of iron chelator had better QoL than the same group in these two countries. Socio-cultural differences between Iran and these countries can be one of the reasons of this discrepancy. Nearly all Iranians are Muslims and based on Islam's teachings, Muslims should be thankful to God in all situations including sickness. Furthermore, Deferasirox is presented by a local pharmaceutical manufacturer in Iran to compete with the original manufacturer of Deferoxamine but in this competition, patients trust more the originator and its footprint remain in mind of the patients.

Mobility, pain and discomfort and usual activity were the items significantly affected by injectable dosage form according to US TTO set. These items are in relation with the difficulties of injection and infusion pump.

Our results suggest that gender has a significant role in health utility score. Based on EQ-5D, females faced with more usual activity problems than males and also had more anxiety and depression. However, studying the comorbidities indicates that, in spite of worse usual activity and depression and anxiety, females had less cardiac problems than males. Based on the studies, the prevalence of depression in non-thalassemic females are more than males.^25^ Furthermore, depression affects usual activity. Therefore, it seems that difference in females' usual activity, anxiety and depression is probably due to gender differences and is independent of thalassemia and its comorbidities. More psychological and usual abnormalities in female patients with thalassemia have also been reported in previous studies.^11^^,^^15^^,^^26^

A combination intake orally and injectable is significantly more frequent in females than males. On the other side, we indicated that there is a relationship between combination use of iron chelators and anxiety and depression. Therefore, higher prevalence of anxiety and depression in combination therapy can be partly due to the gender of the patients.

Comorbidities, as another factor, had meaningful effect on health utility scores. Cardiac complication and diabetes decreased both VAS and total health utility based on EQ-5D and Hepatitis affects only on VAS. Moreover, there is a meaningful relationship between Cardiac complication and mobility and usual activity problems.

As explained before, our sampling was done in one northern, one southeastern and one southwestern province of Iran. The total health utility based on EQ-5D and VAS of patients with thalassemia was similar among these three provinces indicating that, in spite of cultural, socio-economical and geographical differences, the studied populations are uniform enough to be merged. Therefore, the results extracted from this population can be generalized to the whole country. However, socio-economic differences have influenced some of the subscales of QoL. Cardiac complications were more frequent in patients with thalassemia in Sistan-Baluchestan (48.8%). Difficulty in usual activity, pain and discomfort, anxiety and depression were also the main problems of patients in this province. It is likely that these problems are in relation with the high frequency of cardiac complications in this province.

Patient’s age was also one of the effective factors on QoL. Our results suggested that increase in age accompanies with increase in anxiety and depression. Increase in age also accompanied with increase in prevalence of diabetes and hepatitis. Thus, increasing in anxiety and depression seems to be related to diabetes and hepatitis as two main comorbidities of thalassemia in older patients. Furthermore, patients with thalassemia are faced with social challenges. Society usually does not accept these patients properly. Khani et al. showed that 87 percent and 35 percent of patients with thalassemia are single and engage with problems in marriage and occupation, respectively.^26^ These social challenges can be one of the main reasons of anxiety and depression in adult patients. The relation between age groups and health utility was previously studied in other countries. Dahlui et al. showed that increase in age is in coloration with decrease in health utility.^10^ Another investigation studied QoL in patients with thalassemia using SF-36 questionnaire showed that increase in age negatively affect role emotional, role physical, general health, vitality and social functioning.^11^

Our results suggested that in spite of increase in age, patients in the range of 25-30 years have same health utility and most of its subscales with younger patients.

Usual activity of this age range was even better than others. It seems that 25 to 30-year-old patients can manage their problems because of adaptation.

Based on our results, the main problems of Iranian patients with thalassemia were in two subscales of EQ-5D: anxiety and depression and pain and discomfort. They had partly no problem with self care. A pile of evidence substantiates psychological problems as the main problem of patients with thalassemia.^15^^,^^24^^,^^27^^-^^28^

Goulas et al. showed that in comparison with physical functioning, Greek patients with thalassemia face with more psychological problems.^23^ In two other studies conducted by Khani et al. and Musallam et al. patients with thalassemia had least problems in physical functioning but their most problems were in mental health and role-emotional. These patients had no life satisfaction and were subjected to depression and anxiety.^26^^,^^29^ In Khani et al. study, 64.9% of these patients were candidates for receiving psychiatric medical therapies.^26^ Altogether, regarding the importance of psychological support in patients with thalassemia, our study is in the same line with previous studies and emphasizes on the importance of this kind of support along with social support and medical therapies.

## CONCLUSION

 Thalassemic patients have many limitations in their ordinary life which affect their quality of life. By improving the managing method of thalassemia and giving much more social support, we could improve their quality of life.
